# Supplementary material for: Association between the severity of hard-to-treat psoriasis and the prevalence of metabolic syndrome: A hospital-based cross-sectional study in Jakarta, Indonesia
Source: PLoS One. 2024 Apr 29;19(4):e0302391. doi: 10.1371/journal.pone.0302391 (PMC11057762; doi:10.1371/journal.pone.0302391)
Supplement: S1 File — (PDF) [file pone.0302391.s001.pdf]

No.	PATIENT	HOSPITAL	GENDER	AGE (YR)	EDUCATION
1	P01	CMGH	M	72	2
2	P02	CMGH	F	44	2
3	P03	CMGH	M	40	3
4	P04	CMGH	F	31	3
5	P05	CMGH	M	47	3
6	P06	CMGH	M	33	3
7	P07	CMGH	F	25	3
8	P08	CMGH	F	37	2
9	P09	CMGH	M	45	2
10	P10	TGH	F	38	2
11	P11	TGH	M	39	2
12	P12	CMGH	M	39	2
13	P13	TGH	M	61	3
14	P14	TGH	M	66	1
15	P15	TGH	F	47	2
16	P16	TGH	M	30	2
17	P17	TGH	F	40	2
18	P18	CMGH	M	49	2
19	P19	CMGH	M	58	2
20	P20	CMGH	F	24	2
21	P21	PGH	F	23	3
22	P22	PGH	M	42	2
23	P23	CMGH	F	42	2
24	P24	CMGH	F	31	1
25	P25	CMGH	M	63	2
26	P26	PGH	F	45	3
27	P27	CMGH	M	18	3
28	P28	CMGH	F	37	3
29	P29	CMGH	F	72	3
30	P30	CMGH	F	46	2
31	P31	SMH	M	55	3
32	P32	SMH	M	27	3
33	P33	SMH	M	24	3
34	P34	SMH	M	22	3
35	P35	CMGH	F	22	3
36	P36	TGH	M	23	3
37	P37	SMH	F	39	3
38	P38	TGH	F	42	2
39	P39	CMGH	M	62	2
40	P40	PGH	F	59	3
41	P41	PGH	F	58	2
42	P42	CMGH	M	54	2
43	P43	TGH	M	58	3
44	P44	CMGH	M	66	2
45	P45	CMGH	M	22	2

46	P46	CMGH	M	58	2
47	P47	TGH	F	60	2
48	P48	CMGH	F	38	3
49	P49	CMGH	M	31	3
50	P50	CMGH	F	44	3
51	P51	CMGH	M	49	2
52	P52	CMGH	F	46	3
53	P53	CMGH	F	47	2
54	P54	CMGH	M	19	3
55	P55	CMGH	F	48	2
56	P56	CMGH	M	50	2
57	P57	CMGH	M	37	3
58	P58	CMGH	F	53	2
59	P59	CMGH	M	44	2
60	P60	PGH	F	68	3
61	P61	CMGH	F	64	1
62	P62	CMGH	F	25	2
63	P63	CMGH	M	50	1
64	P64	CMGH	F	40	2
65	P65	CMGH	M	72	3
66	P66	CMGH	F	42	3
67	P67	CMGH	M	32	2
68	P68	PGH	F	56	3
69	P69	CMGH	F	53	3
70	P70	CMGH	M	30	2
71	P71	CMGH	M	59	2
72	P72	CMGH	F	38	2
73	P73	CMGH	F	36	3
74	P74	CMGH	M	47	2
75	P75	CMGH	F	58	2
76	P76	CMGH	M	31	3
77	P77	CMGH	F	62	2
78	P78	CMGH	F	34	2
79	P79	CMGH	M	57	3
80	P80	CMGH	F	24	3
81	P81	CMGH	M	48	3
82	P82	CMGH	F	54	3
83	P83	CMGH	F	48	3
84	P84	CMGH	F	38	2

General Information
1: Yes
2: No

Metabolic Syndrome (MetS)
1: Yes
2: No

Educational Level
1 : Low (no formal education - elementary school)
2 : Middle (Junior - High school)
3 : High (Minimal undergraduate)

Employment
1 : Not working/retired
2 : Employee
3 : Self employment
4 : Student

Financing status
1 : National Health Insurance
2 : Other insurance
3 : Out of pocket

Therapy
1: Topical only
2: Combination topical & systemic

Family History with Psoriasis
1: Yes
2: No

Onset of Psoriasis
1. $\leq$ 40 years
2. $>$ 40 years

Hard-to-treat Areas
1: One area involvement
2: 2 areas involvement
3. More than 2 areas involvement

M: Male
F: Female
YR: Years
BMI: Body Mass Index
BP: Blood pressure
TGA: Triglycerides
HDL: High Density Lipoprotein
FBG: Fasting Blood Glucose
BSA: Body Surface Area
PASI: Psoriasis Area Severity Index

EMPLOYMENT	FINANCING	ONSET	DURATION (YR)	HISTORY	THERAPY
1	1	2	10	2	1
3	1	1	7	2	1
3	1	1	6	1	2
3	1	1	6	2	1
3	1	1	29	2	2
3	1	1	17	2	2
3	1	1	10	2	2
1	1	1	14	2	2
2	1	1	20	2	1
1	1	1	19	2	2
2	1	1	1	2	2
3	1	1	4	2	1
3	1	2	13	2	2
1	1	1	36	1	2
1	1	1	10	2	2
3	1	1	4	2	2
1	1	1	1	2	2
3	1	2	4	2	2
3	1	2	9.5	2	2
1	1	1	5	1	2
2	1	1	8	1	2
3	1	1	10	2	2
2	1	1	5	2	2
1	1	1	5	2	1
1	1	2	0.083	2	1
2	1	1	13	2	2
4	1	1	4	2	2
2	1	1	19	2	2
1	1	2	20	2	2
1	1	1	17	2	2
3	3	1	24	2	2
2	3	1	11	2	2
2	3	1	8	2	2
1	3	1	8	2	2
4	1	1	6	2	2
2	1	1	11	2	2
2	3	1	19	1	2
3	1	1	9	2	2
1	1	2	8	1	2
2	1	1	30	2	1
1	1	1	43	1	2
1	1	2	2	2	2
1	1	1	57	1	2
1	1	2	15	1	2
1	1	1	1	2	1

2	1	1	30	2	1
1	1	1	22	2	2
1	1	1	5	2	2
2	1	1	21	2	2
2	1	1	13	2	2
3	1	2	0.75	2	2
2	1	1	18	1	2
2	1	1	9	2	2
4	1	1	0.25	2	1
1	1	1	31	2	2
1	1	1	35	2	2
2	1	1	18	2	1
1	1	2	0.25	2	1
2	1	1	19	2	2
1	1	2	3	1	1
1	3	2	2	2	2
1	1	1	0.083	2	1
2	1	2	4	2	2
1	1	1	7	2	2
1	1	2	4	2	2
1	1	1	24	2	2
2	1	1	18	2	2
1	1	1	20	1	2
2	1	2	4	2	2
2	1	1	5	1	2
2	1	2	6	2	2
1	1	1	12	2	2
2	1	1	12	2	2
3	1	2	4	2	2
2	1	2	14	2	2
2	1	1	10	2	2
1	1	2	17	2	1
1	1	1	21	2	2
3	1	2	12	2	2
4	1	1	4	2	2
2	1	1	13	2	2
1	1	1	18	2	2
2	1	1	12	2	2
2	1	1	1	2	1

HTT AREA	SCALP	FACE	INVERSE	GENITAL	PALMOPLANTAR
3	1	1	1	2	2
3	1	1	1	2	1
3	1	1	1	2	2
2	1	2	1	2	2
2	1	1	2	2	2
2	1	1	2	2	2
3	1	1	1	2	2
2	1	1	2	2	2
3	1	2	1	2	2
3	1	2	1	1	2
3	1	1	1	2	2
3	1	1	1	1	1
2	1	2	1	2	2
3	1	1	1	2	2
3	1	1	1	2	2
3	1	1	2	2	1
1	1	2	2	2	2
3	1	1	1	2	2
3	1	1	1	2	1
3	1	1	1	1	2
3	1	2	1	1	1
3	1	2	1	2	1
2	1	2	2	1	2
3	1	1	2	2	2
3	1	1	1	2	1
3	1	1	1	1	2
3	1	1	1	1	2
3	1	1	1	2	2
3	1	1	2	2	2
3	1	1	1	1	2
3	1	1	1	2	2
3	1	1	1	1	2
3	1	1	2	2	1
3	1	1	1	2	1
3	1	1	1	1	2
3	1	1	1	1	1
3	1	1	2	2	1
2	1	1	2	2	2
2	2	1	2	2	2
3	1	2	1	1	2
3	1	2	1	1	1
3	1	2	1	2	2
2	1	2	2	2	2
3	1	1	1	1	1
3	1	1	1	1	1

3	1	1	1	2	2
3	1	1	1	1	2
2	1	1	2	2	2
3	1	1	1	1	2
3	1	1	1	2	2
3	1	2	1	1	1
3	1	1	1	2	2
3	1	1	1	2	2
3	1	1	1	2	2
3	1	1	1	1	2
3	1	1	1	2	2
3	1	1	1	2	2
3	1	1	1	1	1
3	1	1	1	1	2
3	1	2	1	2	2
3	1	2	1	2	2
1	2	2	1	2	2
3	1	1	1	1	1
3	1	2	1	2	2
3	1	1	2	2	2
3	1	1	1	2	2
3	1	1	1	2	1
3	1	2	1	1	2
3	1	2	1	2	2
3	1	1	1	2	1
3	1	2	1	2	1
3	1	1	2	2	2
3	1	1	1	2	2
3	1	1	1	2	2
3	1	2	1	2	2
3	1	1	1	2	2
3	1	2	1	2	2
3	1	1	1	1	2
1	1	2	2	2	2
3	1	1	1	2	2
2	2	1	2	2	2
3	1	1	1	2	2
3	1	1	2	2	2
3	1	1	2	2	2

NAIL	BSA (%)	PASI SCORE	WAIST CIRCUMFERENCE	HEIGHT	WEIGHT
2	8.5	7.7	100	160	68
2	20	12.4	103	148	70
2	19	18.7	71	170	55
2	10	15	120	165	111
2	36	18.6	80	167	63
2	26	20.6	131	170	119
1	7.5	10	69	158	48
2	15	8.7	96	158	74
1	40	20.4	98	165	70
1	3.5	3.1	84	146	63
1	3.5	3.3	97	167	76
1	43.5	21.4	103	176	94
2	2	1.1	100	178	87.8
1	5.3	3.9	109	179	88
1	25.5	25.8	93	170	75
2	9.5	6.1	74	165	60
2	9.5	11.7	95	150	70
1	40	25.6	83	165	60
1	95	51	90	167	65
2	40	20	78	160	53
1	5.5	7.1	89	144	61
1	5	7.2	101	153	72
2	27	18	105	162	77
1	8	6.4	105.5	152	91.5
1	27	18.6	98	173	170
1	14.5	9.6	81	160	67
1	52	37.4	83	177	70
1	10	10.2	127	163	110
1	1.8	1.6	92	160	60
2	7	7.6	89	157	57
1	31	21.1	122	172	101.75
1	25	9.7	96.5	164	76.05
1	16	7.4	104	173	85.1
1	16	7.4	91.5	162	71.7
2	19.5	16.2	101	167	80
1	4	3.3	70	160	53
2	6	3.3	90	168	65
2	8	8.6	86	150	61
1	2.5	2.9	90	170	73
1	10.5	6.2	112	150	85
2	10	6	80	145	57
2	8	8.6	72	172	58
1	3.3	4.3	96	168	70
1	23	13.2	90	160	60
1	41	21.8	67	168	46

1	20	10.8	106	179	70
1	12.5	7.5	87	153	63
2	8.5	10.4	104	155	80
2	16	9.6	101	170	81
1	13	11.4	94	165	72
1	10	9.5	93	174	78
2	2	2.5	102	152	76
1	10	8.2	90	160	108
2	3	1.7	83	169	61
1	51	27	96	145	58
1	27	10.2	108	170	92
1	22	10.4	109	172	87
2	78	35.2	88	155	65
2	9.5	7	90	170	73
1	23	12.3	110	168	75
1	50	36.3	100	142	57
2	9.5	6.3	84	160	57
1	19	14.4	93.5	165	67
1	3	3.8	78	156	54
1	15.5	10.8	112	167	77
1	6.5	6	98	157	65
1	21.5	9.9	102	165	82
1	6.25	5.9	88	163	57
1	20	14.9	105	158	68
1	21.5	21.6	71	170	57
1	10.5	7.7	105	175	83
1	28	18	107	164	78
2	44	16.9	88	148	67
1	10	7.3	96	179	83
1	15.5	12.4	96.5	149	57
1	20	13.2	108	167	79
1	19	17	113	152	69
2	5	5.4	88	158	67
2	5.3	11.1	120	170	95
2	7	6	70	160	53
1	13.5	14.2	105.5	176	75
2	10	5.6	89	165	73
1	16	12.5	99	160	78
1	8	11.1	88	155	54

BMI	BP	HYPERTENSION TREATMENT	TGA	HDL
26.6	130/86	1	193	43
33.3	153/95	1	132	43
19	114/77	2	68	38
40.8	133/94	1	183	35
22.6	126/80	2	103	38
41.2	144/85	2	64	48
19.2	100/64	2	47	48
29.6	132/95	2	78	43
25.7	144/106	2	101	49
29.6	136/85	1	234	39
27.3	110/79	2	211	36
30.3	138/96	2	64	39
27.5	140/85	2	137	26
27.5	120/76	2	237	29
26	108/70	2	115	42
22	115/73	2	77	48
31.1	122/73	2	152	47
22	97/63	2	73	62
23.3	160/79	1	138	38
20.7	89/57	2	138	34
29.4	110/70	2	28	45
29.9	118/76	2	177	31
29.3	146/80	2	77	55
39.6	132/108	2	196	35
25.1	153/78	1	161	40
26.2	123/77	2	42	52
22.3	111/74	2	54	51
41.8	170/118	1	91	33
23.4	149/77	1	98	44
23.1	113/75	2	62	44
34.1	145/90	1	142	38
31.1	120/80	2	56	50
28.4	108/76	2	77	49
27.1	107/76	2	108	34
28.7	134/90	2	77	43
20.7	118/76	2	60	53
23	120/80	2	135	38
27.1	140/90	2	58	61
25.3	130/101	2	77	61
37.8	110/70	2	141	41
27.1	139/79	1	143	47
19.6	110/68	2	66	41
24.8	135/90	2	101	40
23.4	127/82	2	260	28
16.3	109/71	2	69	36

21.8	157/85	2	184	45
26.9	136/90	1	47	55
33.3	137/89	2	136	38
28	110/72	2	84	41
26.4	153/104	1	146	50
25.8	134/85	1	134	34
32.9	137/92	2	106	47
42.2	131/87	1	113	38
21.4	105/71	2	125	54
27.6	140/100	2	79	47
31.8	147/92	1	161	37
29.4	145/109	1	176	31
27.1	104/72	2	128	42
25.3	132/95	2	76	44
26.6	140/90	1	77	48
28.3	146/90	1	156	49
22.3	111/85	2	51	57
24.6	143/101	1	238	32
22.2	105/70	2	65	66
27.6	150/80	1	244	42
26.4	111/81	2	80	48
30.1	136/97	2	113	44
21.5	126/80	2	185	63
27.2	154/91	1	108	46
19.7	99/66	2	96	47
28.7	147/94	2	277	32
29	142/90	2	95	50
30.6	137/94	1	839	39
26.2	133/91	2	62	45
25.7	128/80	2	71	76
28.3	130/85	2	169	38
29.9	154/83	1	117	36
26.8	119/81	2	93	57
32.9	159/74	1	152	39
20.7	109/61	2	38	67
24.2	138/88	1	372	36
26.8	140/80	2	214	32
30.5	132/84	2	109	34
22.5	107/73	2	169	32

CHOLESTEROL TREATMENT	FBG	FBG TREATMENT	METS	HbA1c LEVEL
1	100	2	1	6.1
1	121	1	1	6.6
2	86	2	2	4.8
1	224	1	1	10.5
1	141	1	1	12.3
2	77	2	2	5.7
2	100	2	2	5.4
2	79	2	1	5.5
2	127	2	1	6
2	78	2	1	5.7
2	79	2	1	5.9
2	85	2	1	5.7
2	77	2	1	5.3
2	97	2	1	6
2	193	1	1	11.6
2	85	2	2	5.2
2	70	2	1	5.1
1	82	2	2	5.4
1	144	1	1	11
2	83	2	2	5.2
2	78	2	2	5.2
1	87	2	1	5.5
2	91	2	2	6
2	339	2	1	14.7
1	163	1	1	7.9
2	87	2	2	5.6
2	82	2	2	5.1
2	87	2	1	5.2
2	102	2	1	6
2	88	2	2	4.4
2	104	2	1	6
2	79	2	2	5.3
2	70	2	2	5
2	79	2	2	5.4
2	88	2	1	5.1
2	93	2	2	5.2
2	64	2	2	5.7
2	90	2	2	5.4
2	98	2	2	5.5
1	119	1	1	6.8
1	89	2	1	5.7
2	84	2	2	6
2	80	2	2	5.2
2	78	2	1	5.3
2	77	2	2	4.9

1	81	2	1	4.4
2	82	2	2	5.4
2	92	2	1	5.5
2	84	2	2	5.1
2	88	2	2	5.1
2	134	1	1	7.3
2	77	2	1	5.4
1	88	2	1	6.2
2	77	2	2	4.6
1	63	2	1	5.0
2	86	2	1	6.1
1	161	1	1	7.8
2	150	2	1	6.2
2	205	2	1	11.6
2	97	2	1	6.2
2	99	2	1	7.8
2	92	2	2	5
2	90	2	1	6
2	81	2	2	5.1
1	115	1	1	7.9
2	82	1	1	4.9
2	69	2	2	6
2	102	2	1	8.1
2	90	2	1	6
2	82	2	2	5.3
1	84	1	1	7.6
1	149	1	1	8.1
1	145	1	1	7.8
2	114	2	1	5.6
1	153	1	1	8.1
1	93	2	1	5.1
2	166	1	1	6.6
1	75	2	1	5.1
1	194	1	1	9.4
2	78	2	2	5
1	88	2	1	5.7
1	159	1	1	7.8
2	87	2	1	5.6
2	88	2	1	4.5

SMOKING	ALCOHOL
2	2
1	1
2	2
2	2
2	2
2	1
2	2
2	2
2	2
2	2
1	2
1	2
2	2
2	2
2	2
2	2
2	2
2	2
2	2
2	2
2	2
1	2
2	2
2	1
1	2
2	2
2	2
2	2
2	2
2	2
2	2
2	2
1	1
1	2
2	2
2	2
2	2
2	2
2	2
2	2
2	2
1	2
1	2
1	2
1	2

2	2
2	2
2	2
2	2
2	2
2	2
2	2
1	2
2	2
2	2
1	2
2	2
2	2
2	2
2	2
2	2
2	2
1	2
2	2
2	2
2	2
1	2
2	2
2	2
1	1
1	1
2	2
2	2
2	2
2	2
2	2
2	2
2	2
2	2
2	2
2	2
2	2
2	2
2	2
