# Supplementary material for: Association between the severity of hard-to-treat psoriasis and the prevalence of metabolic syndrome: A hospital-based cross-sectional study in Jakarta, Indonesia
Source: PLoS One. 2024 Apr 29;19(4):e0302391. doi: 10.1371/journal.pone.0302391 (PMC11057762; doi:10.1371/journal.pone.0302391)
Supplement: Attachment — Submitted filename: Response to Reviewers.docx [file pone.0302391.s002.docx]

Department of Dermatology & Venereology

Faculty of Medicine Universitas Indonesia

Dr. Cipto Mangunkusumo National Hospital

Jalan Diponegoro 71, Jakarta Indonesia

January 4^th^ 2024

Dear Editors,

Subject: Resubmission of the revised manuscript entitled “Association between the severity of hard-to-treat psoriasis and the prevalence of metabolic syndrome: A hospital-based cross-sectional study in Jakarta, Indonesia” (PONE-D-23-19694)

Thank you for your email dated October 23^rd^, 2023 enclosing the reviewers’s comments. We appreciate you and the reviewers for your precious time in reviewing our paper and providing valuable comments. It was your valuable and insightful comments that led to possible improvements in the current version. The authors have carefully considered the comments and tried our best to address each of the requirements. Our responses are given in a point-by-point manner bellow. Changes to the manuscript are shown in the red font

We look forward to your response and hope that the revision in the manuscript will be sufficient to make our manuscript suitable for publication in the PLOS ONE journal.

Sincerely,

Dr. Endi Novianto

Associate Professor of Dermatology and Venereology

Faculty of Medicine Universitas Indonesia - Dr. Cipto Mangunkusumo National Hospital

Tel: 628161309063; Email: [endinoviantodr@gmail.com](mailto:endinoviantodr@gmail.com)

On behalf of all authors

Additional requirements.

1. Please ensure that your manuscript meets PLOS ONE's style requirements, including those for file naming. The PLOS ONE style templates can be found at 
<https://journals.plos.org/plosone/s/file?id=wjVg/PLOSOne_formatting_sample_main_body.pdf> and 
<https://journals.plos.org/plosone/s/file?id=ba62/PLOSOne_formatting_sample_title_authors_affiliations.pdf>

*Author response*: Thank you for your reminder. We will ensure our manuscript meets PLOS ONE’s style requirements.

2. We note that the grant information you provided in the ‘Funding Information’ and ‘Financial Disclosure’ sections do not match.

When you resubmit, please ensure that you provide the correct grant numbers for the awards you received for your study in the ‘Funding Information’ section.

*Author response***:** Thank you for your notification. This is the screen capture of the Financial Disclosure stated in the manuscript.

Below is the screen capture from the funding information

Both statements provide the same grant numbers. We will ensure that we provide the correct grant numbers and information for both sections.

3. We note that you have stated that you will provide repository information for your data at acceptance. Should your manuscript be accepted for publication, we will hold it until you provide the relevant accession numbers or DOIs necessary to access your data. If you wish to make changes to your Data Availability statement, please describe these changes in your cover letter and we will update your Data Availability statement to reflect the information you provide.

*Author response*: Thank you for your notification. We have already share the data through the Fairsharing.org as recommended by PLOS One Journal, but still await for the DOI access. Below the screenshot of the fairsharing web page taken on 4^th^ January 2024.

4. Please remove your figures from within your manuscript file, leaving only the individual TIFF/EPS image files, uploaded separately. These will be automatically included in the reviewers’ PDF.

*Author response*: Thank you for your notification. We will remove all figures from within the manuscript file and upload the individual TIFF/EPS image files separately.

5. Please review your reference list to ensure that it is complete and correct. If you have cited papers that have been retracted, please include the rationale for doing so in the manuscript text, or remove these references and replace them with relevant current references. Any changes to the reference list should be mentioned in the rebuttal letter that accompanies your revised manuscript. If you need to cite a retracted article, indicate the article’s retracted status in the References list and also include a citation and full reference for the retraction notice.

*Author response*: Thank you for your reminder. We will ensure our reference list is complete and correct

**Reviewer's Responses to Questions**

**Comments to the Author**

1. Is the manuscript technically sound, and do the data support the conclusions?

The manuscript must describe a technically sound piece of scientific research with data that supports the conclusions. Experiments must have been conducted rigorously, with appropriate controls, replication, and sample sizes. The conclusions must be drawn appropriately based on the data presented.

Reviewer #1: Yes

Reviewer #2: Yes

*Author Response:* Thank you.

2. Has the statistical analysis been performed appropriately and rigorously?

Reviewer #1: Yes

Reviewer #2: Yes

*Author Response*: Thank you.

3. Have the authors made all data underlying the findings in their manuscript fully available?

The [PLOS Data policy](http://www.plosone.org/static/policies.action#sharing) requires authors to make all data underlying the findings described in their manuscript fully available without restriction, with rare exception (please refer to the Data Availability Statement in the manuscript PDF file). The data should be provided as part of the manuscript or its supporting information, or deposited to a public repository. For example, in addition to summary statistics, the data points behind means, medians and variance measures should be available. If there are restrictions on publicly sharing data—e.g. participant privacy or use of data from a third party—those must be specified.

Reviewer #1: Yes

Reviewer #2: Yes

*Author Response*: Thank you.

4. Is the manuscript presented in an intelligible fashion and written in standard English?

PLOS ONE does not copyedit accepted manuscripts, so the language in submitted articles must be clear, correct, and unambiguous. Any typographical or grammatical errors should be corrected at revision, so please note any specific errors here.

Reviewer #1: Yes

Reviewer #2: Yes

*Author Response*: Thank you.

5. Review Comments to the Author

Please use the space provided to explain your answers to the questions above. You may also include additional comments for the author, including concerns about dual publication, research ethics, or publication ethics. (Please upload your review as an attachment if it exceeds 20,000 characters)

Reviewer #1: General comment.
This study is a replication of numerous earlier, related studies. The writers should succinctly justify the reason for doing it in the Indonesian Population. This can be understood by looking at some of the references in this manuscript, such as references 13, 17, and 19, as well as further uncited work in literature databases. Has ethnicity anything to do with it?

*Author Response*: Thank you for your concern. We have revised the statement to justify the reason for doing the research in the Indonesian Population.

- See line 21-22 *… in Indonesian population.*
- See line 68 – 75:

*Since MetS and psoriasis develop from both genetic and environmental factors, the relationship between psoriasis and MetS may differ amongst different ethnicities or populations due to differences in environmental exposure and genetic background.* *Although most studies have shown link between psoriasis severity and MetS, similar research among Asian psoriatic patients were limited with varying results.* *It is not always feasible to generalize the data and findings beyond populations. Therefore, the aim of the present study was to determine whether hard-to-treat psoriasis severity is associated with the increased prevalence of MetS and which components of MetS play an important role in our Indonesian HTT psoriasis population.*

ABSTRACT
Well-written and summarizes important information in the manuscript.

*Author Response*: Thank you

INTRODUCTION
Lines 67 and 68 the statement “To date no studies have assessed the prevalence of MetS in the HTT psoriasis population and its associated factors”. The statement may be overstated and needs to be revised.

*Author Response*: Thank you for your kind suggestion. It has been revised as requested in the line 67 and 68.

METHODS
Give a reference for the classification of patients using PASI scores that you adopted in this study i.e. (PASI scores ≤ 10 and >10).

*Author Response*: Thank you for your kind suggestion. The PASI score classification was adopted from other similar research by Souza et al. (reference number 2), Itani et al. (reference number 13). We have cited the sentences according to the above references (see lines 100). In addition, study of Nada et al. (reference number 29) has the same cut off but different classification.

RESULTS
1. Lines 126 and 127 “Afterwards the participant also divided into PASI<10 (mild=12, moderate=20, total=42) and >10 groups (severe=42)” Revise the sentence.
2. In the entire manuscript, any numbers with decimal places should be corrected. It appears that (, and.) were mixed up when the number was decimalized.

*Author Response*: Thank you for your kind suggestion.

(1) The sentence has been revised as requested in the line 127.

*Afterwards the participants were also divided into PASI≤10 (mild=12, moderate=20, total=42) and PASI>10 groups (severe=42).*

(2) The numbers with decimal places has been corrected. Decimal is using point (.).

DISCUSSION
Extend the paragraph that describes the study's limitations. Unlike previous writers who employed the case-control design, this study did not use a control group. How did this not compromise the reliability of this study?

*Author Response*: Thank you for your concern. We have extended the paragraph in the line 310 and 314.

Reviewer #2: Abstract
Well summarized and readable and understandable

*Author Response*: Thank you.

Introduction
Well narrowed to the aim of the study

*Author Response*: Thank you.

Material and methods
Data collection
Line 92-93: why only 1st and 2nd author had access to information that identify the participants. Can the author explain

*Author Response*: Thank you for your concern. The 1^st^ and 2^nd^ author had access to all participants’ identity. This is a policy to maintain patient’s confidentiality since the grant is submitted by the first and second author. But the other authors that in charge of treating the patient has an access to his/her patient identity. For example, the author from Tarakan General Hospital have access only to the patient identity from her hospital but not from other hospital.

Line 99-100: How weight and height was measured (the protocol) and what kind of instrument was used? (brand and model). Can the author state.

*Author Response*: Thank you for your kind suggestion. We have include the protocol of how the body weight and height was measured, in the page 8-10 of this file. We used the Tesena TSN 9806 WHS medical digital weighing scale and height measuring (stated in the lines 105 – 108).

*Line 105-107: Body weight and height was measured using the Tesena TSN 9806 WHS medical digital weighing scale and height measuring.*

*Line 107-108: The weight measurement is recorded in kilograms (kg) and height in meters (m).*

Line 105-106: How was the waist circumference measured, protocol used and how Blood pressure was measured and what machine was used the brand and model. Can the author state.

*Author Response*: Thank you for your kind suggestion. We have added the brief explanation of both measurements in the lines 104-105 and 108-109. We used OneMed® OD-235 waist ruler and Omron® BP 1300 blood pressure monitors. The complete protocol can be found in page 9-10 of this file.

*Line 104-105: Using a flexible tape OneMed® OD-235, the waist circumference was measured midway between the iliac crest and the lowest rib.*

*Line 108-109: Blood pressure was measured using Omron® BP-1300 blood pressure monitors.*

Results
Line 126-127: there is calculation error of the data of PASI ≤ 10 (mild 12, moderate 20, total 42). The author needs to revise this statement

*Author Response*: Thank you for your kind notification. We have revised the statement in the line 136: …….*PASI<10 (mild=12, moderate=30, total=42)……*

How did the author take into account the cofounders such as age, life style such as alcohol intake cigarette smoking and physical inactivity as the risk for metabolic syndrome. Can the author explain this?

*Author Response*: Thank you for your kind suggestion. We did multivariate analysis about the confounding factors mention above. In this study, alcohol, and smoking were not confounders. The independence factors associated with MetS are age, severity, and body mass index with Nagelkerke R square 0.553.

Discussion
The findings were well discussed in comparison with previous studies and the pathophysiology of the disease development.

*Author Response*: Thank you.

Conclusion
Well concluded based on the findings of the study.

*Author Response*: Thank you.

6. PLOS authors have the option to publish the peer review history of their article ([what does this mean?](https://journals.plos.org/plosone/s/editorial-and-peer-review-process#loc-peer-review-history)). If published, this will include your full peer review and any attached files.

If you choose “no”, your identity will remain anonymous but your review may still be made public.

*Author Response*: Thank you for the option. Yes, we agree to include it.

**Do you want your identity to be public for this peer review?** For information about this choice, including consent withdrawal, please see our [Privacy Policy](https://www.plos.org/privacy-policy).

Reviewer #1: **Yes:** Oscar Mbembela

Reviewer #2: No

*Author Response*: Thank you to both reviewers

**Protocol Weight Measurement**

1. Weight is measured with a calibrated, electronic digital scale. We use Tesena® TSN 9806 WHS medical digital weighing scale and height measuring.
2. The scale should be placed on a hard-floor surface (not on a floor which is carpeted or otherwise covered with soft material).
3. Identify the subject and explain the procedure.
4. The weight measurement is recorded in kilograms (kg).
5. Place a clean paper towel on the scale foot stand. Zero the scale.
6. The digital LED readout should show 000.00 before weighing a sampled person. If it does not, press the zero key on the scale to zero.
7. Participants are asked to remove their heavy outer garments (jacket, coat, trousers, skirts, etc.), shoes, and any other articles of clothing or jewelry. Participants should be in minimal underclothing. They are asked to wear an examination gown (light clothes) and to remove objects such as cell phones or wallets from their hands or pockets.
8. Participants should be weighed at the same time of the day, if possible, after voiding.
9. The health provider directs participants to stand in the center of the scale platform with hands at their sides and looking straight ahead. They should stand still with weight evenly distributed on both feet.
10. Read and record the weight accurately to the resolution of the scale (the nearest 0.1 kg)
11. Calibration should occur at the beginning and end of each examining day. For calibrating an electronic scale, follow the instructions of the specific scale. Note that the reading of an electronic scale depends on the gravity of each location. Therefore its calibration is particularly important whenever a new examination site is set up.

**Protocol Height Measurement**

1. The height was measured using the Tesena® TSN 9806 WHS medical digital weighing scale and height measuring. The height rule is taped vertically to the hard flat wall surface with the base at floor level. The floor surface next to the height rule must be hard.
2. The participants asked to remove hair ornaments, jewelry, buns, braids from the top of the head, and shoes.
3. The participant is asked to stand with his/her back to the height rule. The back of the head, back, buttocks, calves and heels should be touching the stadiometer, feet together. The top of the external auditory meatus (ear canal) should be level with the inferior margin of the bony orbit (cheek bone). The participant is asked to look straight.
4. The head piece of the stadiometer or the sliding part of the measuring rod is lowered so that the hair (if present) is pressed flat.
5. Height is recorded to the resolution of the height rule (i.e. nearest millimeter/half a centimeter). If the participant is taller than the measurer, the measurer should stand on a platform so that he/she can properly read the height rule.
6. If the person is taller then the maximum height of the stadiometer, the self reported height is acceptable and recorded on the collection form.
7. Calibration occur at the beginning and end of each examination day, the height rule should be checked with standardized rods and corrected if the error is greater than 2 mm.

**Protocol Blood Pressure Measurement**

| Conditions | - Quiet room with comfortable temperature. |
| --- | --- |
|  | - Before measurements: Avoid smoking, caffeine and exercise for 30 min; empty bladder; remain seated and relaxed for 3–5 min. |
|  | - Neither patient nor staff should talk before, during and between measurements. |
| Positions | - Sitting: Arm bare and resting on table with mid-arm at heart level; back supported on chair; legs uncrossed and feet flat on floor (Figure 1). |
| Device | - Validated electronic (oscillometric) upper-arm cuff device. Lists of accurate electronic devices for office, home and ambulatory BP measurement in adults, children and pregnant women are available at [www.stridebp.org](http://www.stridebp.org). (We used Omron HBP-1300) |
|  | - Alternatively use a calibrated auscultatory device, (aneroid, or hybrid as mercury sphygmomanometers are banned in most countries) with 1st Korotkoff sound for systolic blood pressure and 5th for diastolic with a low deflation rate. |
| Cuff | - Size according to the individual’s arm circumference (smaller cuff overestimates and larger cuff underestimates blood pressure). |
|  | - For manual auscultatory devices the inflatable bladder of the cuff must cover 75%–100% of the individual’s arm circumference. For electronic devices use cuffs according to device instructions. |
| Protocol | - At each visit take 3 measurements with 1 min between them. Calculate the average of the last 2 measurements. If BP of first reading is <130/85 mm Hg no further measurement is required. |
| Interpretation | - Blood pressure of 2–3 office visits ≥140/90 mm Hg indicates hypertension. |

(Based on 2020 International Society of Hypertension Global Hypertension Practice Guidelines – Unger T., et al. Hypertension. 2020;75:1334–1357)

Figure 1. How to measure blood pressure

**Protocol Waist Circumference**

1. The World Health Organization and the International Diabetes Federation (IDF) suggest measuring WC in the horizontal plane midway between the lowest ribs and the iliac crest (WC-mid).
2. Using a flexible tape OneMed® OD-235, the waist circumference was measured midway between the iliac crest and the lowest rib.
3. The waist circumference measurement is recorded in centimeter (cm).
4. Cut-off values for central obesity for Asians of 90 cm WC-mid for males and 80 cm WC-mid for females based on the recommendation of Asia-Pacific Perspective: Redefining Obesity and its Treatment Conference in 2000.
